# Gene-expression profiling of calves 6 and 9 months after inoculation with *Mycobacterium avium* subspecies *paratuberculosis*

**DOI:** 10.1186/s13567-014-0096-5

**Published:** 2014-10-02

**Authors:** Joel David, Herman W Barkema, Le Luo Guan, Jeroen De Buck

**Affiliations:** Department of Production Animal Health, University of Calgary, 3330 Hospital Drive, T2N 4N1 Calgary, AB Canada; Department of Agriculture, Food & Nutritional Science, University of Alberta, Agriculture/Forestry Centre, Edmonton, AB Canada

## Abstract

Early detection of Johne’s disease (JD) caused by *Mycobacterium avium* subspecies *paratuberculosis* (MAP) is essential to reduce transmission; consequently, new diagnostic techniques and approaches to detect MAP or markers of early MAP infection are being explored. The objective was to identify biomarkers associated with MAP infection at 6 and 9 months after oral inoculation. Therefore, gene expression analysis was done using whole blood cells obtained from MAP-infected calves. All MAP-inoculated calves had a cell-mediated immune response (IFN-γ) to Johnin PPD specific antigens, and 60% had an antibody response to MAP antigens. Gene expression analysis at 6 months after inoculation revealed downregulation of chemoattractants, namely neutrophil beta-defensin-9 like peptide (BNBD9-Like), S100 calcium binding protein A9 (s100A9) and G protein coupled receptor 77 (GPR77) or C5a anaphylatoxin chemotactic receptor (C5a2). Furthermore, BOLA/MHC-1 intracellular antigen presentation gene was downregulated 9 months after inoculation. In parallel, qPCR experiments to evaluate the robustness of some differentially expressed genes revealed consistent downregulation of BOLA/MHC-I, BNBD9-Like and upregulation of CD46 at 3, 6, 9, 12, and 15 months after inoculation. In conclusion, measuring the expression of these genes has potential for implementation in a diagnostic tool for the early detection of MAP infection.

**Electronic supplementary material:**

The online version of this article (doi:10.1186/s13567-014-0096-5) contains supplementary material, which is available to authorized users.

## Introduction

*Mycobacterium avium* subsp. *paratuberculosis* (MAP) causes Johne’s disease (JD) in ruminants, characterized by a chronic granulomatous intestinal infection [1]. Infections with MAP are prevalent worldwide in ruminants, but are also suggested to play a role in Crohn’s disease in humans [2]. Both are driving the increasing interest in MAP and JD. The primary route of transmission is fecal-oral, with infected cattle typically remaining subclinical for an extended interval [3]. In the end stages of JD, MAP-infected cows have chronic diarrhoea and emaciation, leading to early culling. Infection with MAP is estimated to cost the Canadian dairy industry $15 to 20 M annually [4].

Although MAP was discovered as the causative organism of JD early in the 19^th^ century, there are still numerous gaps in our knowledge with regard to JD pathogenesis and diagnostic methods that allow early detection of MAP are lacking. Existing diagnostic tools and strategies are inadequate, due to lack of sensitivity to detect MAP infection during early subclinical stages [5-8] when diagnostic test like ELISA and fecal culture fail to detect infection or have poor sensitivity. Hence, novel biomarkers are considered as alternatives for early identification of the infection. Translation genomics and protein arrays have been applied to identify biomarkers for early detection of JD [9-11]. Potential biomarkers for diagnosis include genes involved in host stress and immune response to the disease, suggesting that understanding the pathophysiology of MAP infection is crucial in identifying specific biomarkers. Moreover, a practical biomarker needs to be specific, robust and easily measurable without invasive procedures [10]. In a previous study, several putative biomarkers of early (3 months after inoculation) MAP infection were found with particular roles in the immune response [12]. Furthermore, the importance of dose of infection on the discovery of biomarkers was also apparent in that study. The objectives of the current study were to identify potential transcriptional biomarkers for MAP infection at 6 and 9 months stage of inoculation and to confirm the influence of infective dose on these transcripts. Furthermore, we aimed to analyse expression levels of select gene targets over a 15 months period after inoculation to validate their potential use as early diagnostic biomarkers.

## Materials and methods

### Animals

Selection, nutrition, health and husbandry of the animals and the design of the MAP infection experiment have been reported [12,13]. In short, Holstein-Friesian bull calves were procured from Alberta (Canada) dairy farms with zero MAP fecal culture prevalence and a low (< 5%) MAP sero-prevalence. Five calves were orally inoculated with a high dose (HD) [14] of MAP, 5 with a low dose (LD), and another 5 were kept as non-inoculated controls; the bacteria were a virulent cattle type MAP strain isolated from a clinical Alberta JD case (Cow 69). This isolate has an identical BamHI, PvuII and PstI IS900 – RFLP profile as the reference strain K10 recommended for use in infection trials [15]. Calves were inoculated at 2 weeks of age on 2 consecutive days, with either 5 × 10^9^ CFU (HD) or 5 × 10^7^ CFU (LD). The animal care protocols were approved by the Animal Care Committee of the University of Calgary (M09083).

### MAP exposure assays

Both control and MAP-inoculated calves were tested every month for exposure to MAP. Humoral response was determined with a commercial antibody ELISA (Pourquier ELISA™; Institut Pourquier, Montpellier, France) as previously described in [16] whereas the cell-mediated immune response used an IFN-γ release assay. For the latter, whole blood was incubated in a 24-well plate overnight with Johnin PPD and post-incubation supernatants were used for BOVIGAM® IFN-γ ELISA (Prionics, Schlieren-Zurich, Switzerland). Liquid MAP culture (TREK para-JEM®; TREK Diagnostic Systems, Cleveland, OH, USA) was performed as described previously in [17] on fecal samples collected weekly, starting in the first month after inoculation and thereafter, monthly until necropsy. All calves were euthanized at 17 months of age. Macroscopic and histological lesions were assessed and bacterial culture was done on numerous tissues [13].

### Sample collection and preparation

Every 3 months, whole blood was collected from HD, LD and control calves in PAXgene® Blood RNA system tubes (PreAnalytix GmbH, Hombrechtikon, Switzerland). Total RNA was extracted from whole blood using the PAXgene® blood miRNA kit (PreAnalytix GmbH) as per kit protocol. The quality of total RNA was determined using an RNA integrity number (Agilent RNA 6000 NanoChip on 2100 Bioanalyzer, Agilent Technologies, Santa Clara, CA, USA). Moreover, 5–10 μg of extracted total RNA was processed using RNeasy Plus Micro kit (Qiagen, Mississauga, ON, Canada) to remove any genomic DNA carryover. The RNA was quantified using NanoDrop ND-1000 (NanoDrop Technologies, Wilmington, DE, USA). Although total RNA was extracted from whole blood collected from trial calves at 3, 6, 9, 12 and 15 months, only total RNA from 6 and 9 months after MAP exposure was used for gene expression analysis by microarray.

### Hybridization

Biotin-labelled antisense RNA (aRNA) was prepared from 100 ng of genomic DNA purified from total RNA using a GeneChip® 3’-IVT express kit (Affymetrix, Santa Clara, CA, USA). Furthermore, 12 μg of biotin-labelled aRNA was fragmented (35–200 nt) at 95 °C for 35 min for hybridization. Fragmented biotin-labelled aRNA were hybridized to an Affymetrix® GeneChip® Bovine genome Array at 45 °C for 16–18 h. Arrays were stained with streptavidin-phycoerythrin and washed using Affymetrix® GeneChip Fluidics 450 following manufacturer’s protocol and scanned with an Affymetrix® GeneChip Scanner 3000 7G System at the Southern Alberta Microarray Facility (Calgary, AB, Canada).

### Gene expression statistical analysis

Gene expression profiles were generated as CEL files using GeneChip® Command Console® Software (AGCC), with GeneSpring™ Gx (Agilent Technologies, Santa Clara, CA, USA) used for statistical analyses. Raw data were normalized and summarized using a robust multichip averaging (RMA) algorithm and Probe Logarithmic Intensity ERror (PLIER) algorithm, as reported [12]. Both RMA and PLIER algorithms employ quantile normalization; the former uses perfect match (PM) to correct the background fluorescence intensity, whereas the latter uses both PM and mismatch probes from the error correction. Gene transcripts were filtered based on probe expression levels (default settings for low and high were 20 and 80%, respectively), with coefficient variation ≤ 50% (part of quality control). In addition, quality control passed probe sets were analysed using ANOVA to check for differentially expressed probe sets among HD, LD and control calves. The Benjamini-Hochberg FDR correction method was used to obtain reliable gene expression data. Finally, 1.5-fold change analysis was done to determine fold change of differentially expressed probes. Hierarchical clustering and Principal Component Analysis (PCA) were performed on the entities designated differentially expressed (by ANOVA) to determine the significant differential gene expression profile between analysed groups.

### Systems biology

An additional analysis was performed with ANOVA (*p* ≤ 0.10) on both RMA and PLIER to obtain entities for systems biology analysis. Ingenuity® Systems Pathway Analysis (IPA; Ingenuity Systems, Redwood City, CA, USA) was used to identify biological relevance, annotate and predict molecular and cellular functions of each gene and identify their involvement in biological processes. Differentially expressed probesets (based on ANOVA) were submitted as Affymetrix® GeneChip® Bovine Genome Array gene set and annotated using bovine genome array databases available on IPA.

Significance of relationship to a functional category or pathway was assigned by calculating the ratio between total molecules in that function by the number of molecules from the dataset. Significance was also estimated by a right-tailed Fisher’s exact test (relationships with *p* ≤ 0.05 were considered significant). In addition, Z-scores were calculated for upstream and downstream analyses and tested for significance to place differentially expressed transcripts in the context of functional categories and pathways.

### Reverse transcription for qPCR

Total RNA, extracted from the blood of the calves at 3, 6, 9, 12 and 15 months after infection, was used to prepare cDNA for qPCR analysis. Reverse Transcription (RT) reaction was carried out using The Quantitect® Reverse transcription kit (Qiagen, Mississauga, ON, Canada). For this, 1 μg of genomic DNA purified total RNA was used to prepare cDNA, which involved a genomic DNA elimination reaction and an RT reaction at 42 °C for 10 and 30 min, respectively. The RT mix included oligo-dT and random primers and omniscript and sensiscript reverse transcriptase enzymes to synthesize cDNA. For the post RT-reaction, all cDNA samples was diluted to 100 ng/μL and stored at −20 °C to be used for qPCR validation.

### Real-time qPCR

Based on fold change and relevance to the study, 6 genes from 6 months after infection gene expression data and 5 from 9 month gene expression data were chosen for qPCR validation. Intron-spanning primers for these genes were designed using the Primer3 Version 0.4.0 online tool. Primers were synthesized at University of Calgary DNA synthesis lab (Calgary, AB, Canada); primer sequences and information are shown (Tables 1 and 2). Based on previous publications [18,19] and geNorm analysis done using qBase^PLUS^ (Biogazalle, Zwijnaarde, Belgium), GAPDH was used as a reference gene for normalization. For these studies, 100 ng of cDNA was used for all samples and experiments were done using Qiagen quantitect SYBR green reagents (Qiagen, Mississauga, ON, Canada) on CFX96™ Real-Time PCR detection system. The MIQE guidelines were adopted for qPCR confirmation assays, as recommended [20]. Prior to gene expression analysis, repeatability and efficiency for each primer was analysed using Bio-Rad CFX Manager™ 2.0 (Bio-Rad, Mississauga, ON, Canada) as part of the primer standardization procedure. Pooled RNA samples were used as non-reverse transcription (NRT) control to measure genomic DNA carry over. Real-time PCR amplification in this study involved initial enzyme activation at 95 °C for 15 min and 45 cycles of 95 °C for 10 s, 15 s at 60 °C, and 15 s at 72 °C extension step. Melt curve analysis was done at the end of amplification to ensure product specificity. Cycles of threshold values obtained by gene expression qPCR assays were exported in Excel format using Bio-Rad CFX Manager™ 2 for statistical analysis. Calculations for gene expression analysis were done in Excel, using the 2^-δδCT^ method; log2 fold changes obtained were then analysed by ANOVA (*p* ≤ 0.05) with Tukey post hoc tests done using GraphPad Prism version 5.0c (La Jolla, CA, USA) for Mac OS X.

**Table 1:** **qPCR primers used for 6 month gene targets**

**Target**	**Primer sequence (5′-3′)**	**Amplicon size**	**Ensembl gene ID**
BOLA	ACATGGAGCTTGTGGAGACC	283 bp	ENSBTAG00000002069
	CTTGCAGCCTGGGTGTAGAT		
BNBD9-Like	TCTTCCTGGTCCTGTCTGCT	106 bp	ENSBTAG00000047740
	ATCTGTCTCGTGCGTCCAG		
ALOX15	CCACCAAGGATGTGACACTG	251 bp	ENSBTAG00000011990
	GTATTCGTAGGGCCAGTCCA		
ALOX5AP	TTCTCTGCAGCCAAGTTCCT	251 bp	ENSBTAG00000013201
	GAGAAGGAGAGGGGAGATGG		
S100A9	GTCACAAATGGAAAGCAGCA	238 bp	ENSBTAG00000006505
	GGCCACCAGCATAATGAACT		
GPR77	CCGAAACTGTGCACTCAAGA	222 bp	ENSBTAG00000037735
	GCCGAGAGAATTGTTCTCCA

**Table 2:** **qPCR primers used for 9 month gene targets**

**Target**	**Primer sequence (5′-3′)**	**Amplicon size**	**NCBI Accession number**
BOLA	GAACTACCTGGAGGGCGAGT	229 bp	ENSBTAG00000019386
	GGTTTCCACAAGCTCCATGT		
CCR7	CCCTTCTCGTCATTTTCCAG	158 bp	ENSBTAG00000015133
	GGAGTACATGATCGGGAGGA		
IGSF6	TTTCCCAACTCAAAGCAACC	238 bp	ENSBTAG00000018869
	TACGCCGAGCACTCTTTTTC		
IL4R	GTGTGCGTGTCCTGCTACAT	202 bp	ENSBTAG00000001602
	GTAAACAGGGCAGGAGCTTG		
TEX261	CACCTACATGATTGGCGTTG	226 bp	ENSBTAG00000002105
	GAAGAACGCAAACGGGATTA		

Additional qPCR reactions were done on selected genes (BOLA, CD46 and BNBD9-like) on samples obtained at 3, 6, 9, 12 and 15 months to characterize expression profiles of these genes over time. Two-way ANOVA (*p* ≤ 0.05) was performed on the longitudinal gene expression analysis to determine statistical significance for the expression profiles of these genes.

## Results

### MAP exposure test results

All MAP-inoculated calves (HD and LD) became and remained positive for Johnin PPD-specific IFN-γ response within 3 months after inoculation, whereas all control calves remained negative for the duration of the study. All MAP-inoculated calves, except one LD calf, shed MAP at least once during the 17-month follow-up and had MAP-positive intestinal tissues at necropsy. Furthermore, MAP was detected in fecal samples from one control calf by PCR, but no viable MAP was not cultured.

Control calves remained ELISA-negative, whereas 4 out of 5 (80%) HD MAP-inoculated cattle and 2 out of 5 (40%) LD MAP-inoculated cattle became ELISA-positive.

### Gene expression 6 months after inoculation

**Figure 1:**
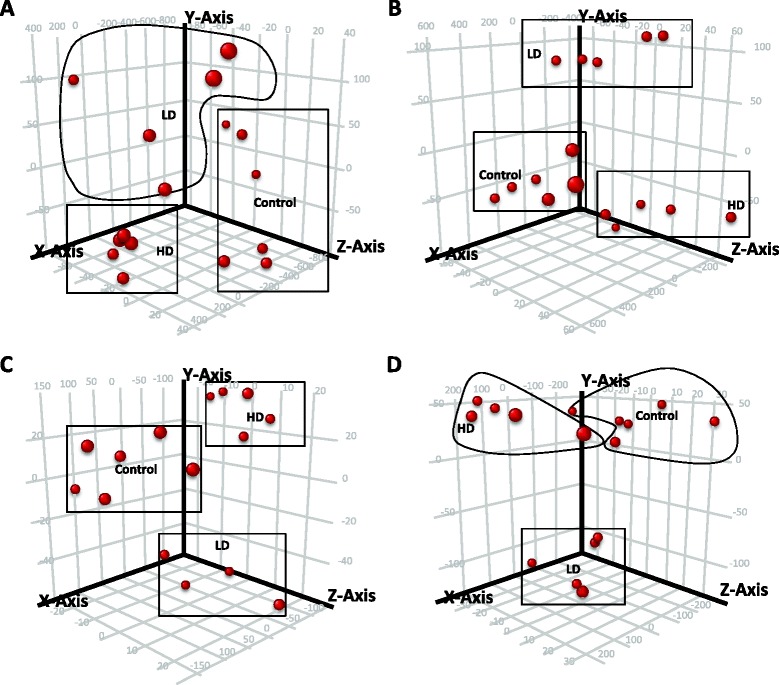
**Principle Component Analysis on entities differentially expressed.** Differentially expressed entitites between treatment groups were identified using ANOVA (*p* ≤ 0.05) at 6 months **(A-B)** and 9 months **(C-D)** after inoculation using RMA or PLIER algorithms respectively. A distribution for all 16 animals obtained using three principal components is shown in a 3D space. At 6 months, the percent variances of the three components by RMA were 54.88, 11.69 and 6.5%, and by PLIER 59.01, 10.01 and 4.17%. At 9 months, the percent variances of the three components were by RMA were 46.83, 14.94 and 5.52%, by PLIER 50.72, 16.0 and 5.99%.

Gene expression analysis, using RMA and PLIER algorithm summarized entities, identified 101 transcripts that were differentially expressed, based on ANOVA using RMA and 186 transcripts using PLIER algorithm. Likewise, 1.5 fold-change analyses revealed 37 transcripts differentially expressed in RMA and 21 transcripts using the PLIER algorithm. In addition, PCA done on transcripts obtained by ANOVA resulted in clear separation of the samples into HD, LD and control groups (Figure 1A and B). In the overall gene expression profile, both HD and LD calves had 60% of their differentially expressed genes upregulated compared to the control. Furthermore, HD and LD calves had opposite expression profiles for 50% of their differentially expressed genes. Using hierarchical clustering on MAP exposure (HD, LD and control), control calves clustered in a separate branch from HD and LD with a centroid Euclidean distance of 0.32. HD and LD calves clustered as sub clusters under a same branch with a centroid Euclidean distance of 0.17 from each other. Additional ANOVA analysis with *p* ≤ 0.10 on both RMA and PLIER summarized entities resulted in 1702 entities for systems biology analysis. A complete list of the differentially expressed genes is included (Additional file 1).

Arachidonate 15-lipoxygenase (ALOX15), Arachidonate 5-lipoxygenase activating protein (ALOX5AP), neutrophil beta-defensin-9 like peptide (BNBD9-Like), S100 calcium binding protein A9 (s100A9) and G protein coupled receptor 77 (GPR77) or C5a anaphylatoxin chemotactic receptor C5a2 were downregulated in both HD and LD calves (Table 3).

**Table 3:** **Fold change comparison between microarray and real-time qPCR for all gene targets between HD, LD and control groups at 6 month after MAP infection**

	**Microarray fold change**			**Real-time qPCR fold change**					
**Gene**	**High vs. control**	**Low vs. control**	**High vs. low**	**High vs. control**	***p*** **-value**	**Low vs. control**	***p*** **-value**	**High vs. low**	***p*** **-value**
BOLA	0.83	0.88	0.94	0.18	< 0.001	0.58	< 0.01	0.32	< 0.01
BNBD9-Like	0.69	0.63	1.01	0.56	< 0.01	0.19	< 0.001	0.33	< 0.01
ALOX5AP	0.54	0.68	0.81	0.58	< 0.01	1.56	< 0.01	0.38	< 0.01
ALOX15	0.35	0.83	0.42	0.35	< 0.01	0.74	< 0.05	0.48	< 0.01
GPR77	0.68	0.66	1.02	0.58	< 0.05	0.56	< 0.05	0.91	NS
S100A9	0.64	0.64	0.99	0.39	< 0.05	0.61	< 0.05	0.64	< 0.05

### Systems biology analyses for genes differentially expressed at 6 months after inoculation

Of the 1702 entities submitted to IPA, 1066 were used for systems biology analyses. Downstream function analysis revealed activation of lymphocyte movement and its migration, migration of mononuclear leukocytes and intracellular infection of the cells. In addition, IPA predicted inhibition of phagocytosis by antigen presenting cells, phagocytosis by macrophages, migration of granulocytes, immune response by macrophages, necrosis, apoptosis and downregulation of genes that would inhibit growth. All these predictions had a significant Z-score (either ≥ 2 signifying activation or ≤ −2 indicating downregulation). Pathway analysis indicated that the differentially expressed genes had roles in several biological pathways; the top 5 canonical pathways included the superpathway of Inositol phosphate compounds, D-myo-inositol (1,4,5,6)-Tetrakisphosphate biosynthesis, D-myo-inositol (3,4,5,6)-Tetrakisphosphate biosynthesis, D-myo-inositol-5-phosphate metabolism and 3-phosphoinositide degradation. JAK-STAT signalling pathway, a key regulator of immune response, was among the top canonical pathways (14^th^ rank). The complete list of the canonical pathways influenced by our differentially expressed genes is shown (Additional file 2).

Upstream analysis predicted activation of CD40 ligand (CD40L), CD24, matrix metallopeptidase 14 (membrane-inserted) (MMP14) and TNF family proteins. Transforming growth factor beta 1 (TGF-beta1), several micro RNA’s (miRNA) were predicted to be inhibited based on expression levels of molecules differentially expressed among the 3 groups. A list of all the upstream regulators and their direction of activation along with Z-score is shown (Additional file 3).

### Gene expression 9 months after inoculation

Analysis of gene expression using RMA identified 31 and 12 transcripts (based on ANOVA and 1.5 fold-change analyses, respectively). Furthermore, ANOVA and 1.5 fold-change analyses using PLIER algorithm detected 49 and 3 transcripts, respectively, that were differentially expressed. Furthermore, PCA analysis on entities differentially expressed by ANOVA (*p* ≤ 0.05) resulted in clear separation of HD, LD and control groups (Figure 1C and D). In the overall gene expression profile, HD calves had 55% of their transcripts upregulated compared to control calves, whereas the LD group had 86% of transcripts upregulated compared to controls. Hierarchical clustering on conditions (HD, LD and control groups) of differentially expressed entities had HD calves clustered in a separate branch from control and LD groups (centroid Euclidean distance of 0.25). Control and LD calves were subclusters under the same branch (centroid Euclidean distance of 0.20). Additional ANOVA analysis with *p* ≤ 0.10 performed on both RMA and PLIER algorithm summarized entities resulted in 408 differentially expressed transcripts which were used for systems biology analyses. A complete list of differentially expressed transcripts with their fold-changes is shown (Additional file 4).

Nine months after inoculation, bovine leukocyte antigen (BOLA), interleukin 4 receptor (IL4R), chemokine (C-C motif) receptor 7 and testis expressed 261 (TEX261) genes were downregulated in HD and LD calves. Immunoglobulin super family member 6 (IGSF6), ribosomal protein L15 (RPL15) were upregulated in both LD and HD calves.

### Systems biology analyses for genes differentially expressed at 9 months after inoculation

Of the 408 entities submitted to IPA, 275 entities were used for systems biology analyses. Downstream function analysis predicted differentially expressed transcripts to activate autophagy, activation of antigen presenting cells, cytotoxicity of T-lymphocytes, and organisation of the cytoplasm for cell replication. Downstream analysis also predicted inhibition of apoptosis, generation of T-lymphocytes and cell spreading. However, these functions lacked significant Z-scores.

The top 5 canonical pathways included acute myeloid leukaemia signalling, methionine degradation I (to homocysteine), cysteine biosynthesis III (Mammalia), ERK5 signalling and Neurotrophin/TRK signalling. The IL-4 signalling pathway, involved in Th2 response, was the 9^th^ canonical pathway. A complete list of all canonical pathways in which differentially expressed genes were predicted to be involved is shown (Additional file 5). Upstream analysis predicted inhibition of several miRNAs, hypoxia inducible factor 1, alpha subunit (HIF1A), chemokine (C-X-C motif) ligand 12, forkhead box O3 (FOXO3) and myelocytomatosis viral oncogene homolog (Avian) (MYC) and predicted few miRNAs to be activated. A list including complete upstream analysis results is shown (Additional file 6).

### Overlap of affected pathways at 3, 6 and 9 months after inoculation

Differentially expressed genes were found in a total of 476 canonical pathways, at either 3 [12], 6 and 9 months after inoculation, with 55%, 73% and 56% of these pathways affected at the respective time points. A total of 89 (19%) of these pathways were affected at every of the three time points, and 36%, 43% and 24% overlapped at respectively 3 and 6, 6 and 9 and 3 and 9 months after inoculation. At those respective times, only 24%, 17% and 12% of the pathways were unique to these time points.

### Real-time qPCR confirmation microarray data

**Figure 2:**
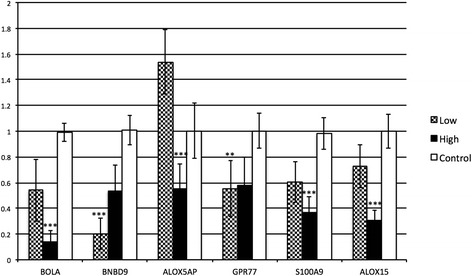
**qPCR validation of gene expression data at 6 month after inoculation.** The bar diagram shows the comparison of fold changes (Y axis) of high and low dose groups compared to control group for each of the gene targets (X axis). ANOVA with Tukey post-hoc test was used for this analysis, with ** indicating significant difference with *p* ≤ 0.01 and *** indicating significant differences with *p* ≤ 0.001.

**Figure 3:**
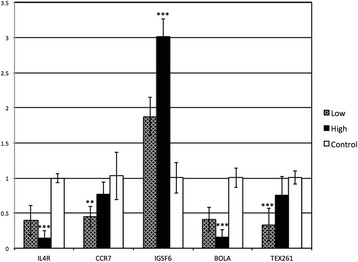
**qPCR validation of gene expression data at 9 month after inoculation.** The bar diagram shows the comparison of fold changes (Y axis) of high and low dose groups compared to control group for each of the gene targets (X axis). ANOVA with Tukey post-hoc test was used for this analysis, with ** indicating significant difference with *p* ≤ 0.01 and *** indicating significant differences with *p* ≤ 0.001.

To validate microarray findings, 6 gene targets from the 6 months after inoculation gene expression data and 5 gene targets from 9 months after inoculation gene expression data were chosen to be verified using real-time qPCR. Genes for qPCR validation were chosen based on the relevance of the transcripts to JD and their fold change. Genes selected to confirm gene expression data at 6 months after inoculation were BOLA, BNBD9-Like, ALOX-15, ALOX5AP, GPR77 and s100A9. Similarly, genes selected for qPCR confirmation of gene expression data at 9 months after inoculation were BOLA, TEX261, CCR7, IL4R and IGSF6. Quantitative gene expression data obtained for these genes using qPCR was in agreement with microarray data. Real-time qPCR confirmation of the differentially expressed genes at 6 and 9 months after MAP inoculation are shown (Figures 2 and 3, respectively). A comparison of microarray expression data and qPCR expression data for the confirmed genes obtained 6 and 9 months after MAP inoculation are presented (Tables 3 and 4).

**Table 4:** **Table illustrating the fold change comparison between microarray and real-time qPCR for all gene targets between HD, LD and control groups at 9 months post MAP-infection**

	**Microarray fold change**			**Real-time qPCR fold change**					
**Gene**	**High vs. control**	**Low vs. control**	**High vs. low**	**High vs. control**	***p*** **-value**	**Low vs. control**	***p*** **-value**	**High vs. low**	***p*** **-value**
IL4R	0.88	0.93	0.95	0.21	< 0.01	0.48	< 0.01	0.46	< 0.05
CCR7	0.81	1.01	0.73	0.72	< 0.05	0.49	< 0.05	0.67	< 0.05
IGSF6	1.88	1.23	1.53	3.02	< 0.01	2.72	< 0.01	1.37	< 0.01
BOLA	0.38	0.41	1.08	0.22	< 0.01	0.47	< 0.05	0.45	< 0.05
TEX261	0.74	0.81	0.90	0.71	< 0.01	0.39	< 0.01	1.52	< 0.05

### Longitudinal qPCR expression analysis of CD46, BOLA and BNBD9-Like genes

**Figure 4:**
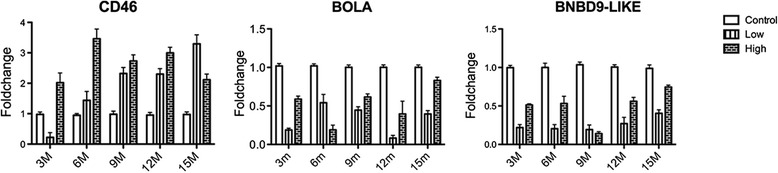
**Longitudinal expression profiles of CD46, BOLA, BNBD9.** The bar diagram shows the comparison of fold changes (Y axis) of high and low dose groups compared to control group for each time point (X axis). Two-way ANOVA with Bonferroni post-hoc test was used for this analysis.

Based on the gene expression studies by microarray analysis at 3, 6 and 9 months after MAP inoculation, CD46, BOLA and BNBD9-Like were differentially expressed at all 3 measured time points. Longitudinal expression profiling of these genes at 3, 6, 9, 12 and 15 months after MAP inoculation by qPCR, demonstrated that CD46 was continuously upregulated in both HD and LD calves from 3 to 15 months after MAP inoculation onwards, except for 3 months after MAP inoculation for LD calves. Both BOLA and BNBD9-Like genes were downregulated in MAP-inoculated calves (HD and LD) at time points 3 to 15 months after inoculation. Two-way ANOVA done on the longitudinal expression values for all 3 genes revealed significant differences (*p* ≤ 0.01). The relative expression of CD46, BOLA and BNBD9-Like genes across time are shown in Figure 4.

## Discussion

Inoculation with MAP (HD or LD) resulted in a chronic infection in all calves, confirmed by fecal shedding, antibody ELISA [16] and positive culture of intestinal tissues [17]. However, one LD calf merely had a repeated positive interferon-gamma test as an indication of exposure to MAP.

Early diagnosis of MAP, similar to diagnosis of *Mycobacterium tuberculosis*, is challenging and largely dependent on detection of cellular immune responses, due to low sensitivity for other tests [21,22] at this stage of infection. Regardless, detection of cellular immune responses is undermined by test complexity and specificity, especially during the early stages of the disease [8,23,24]. Hence, new biomarkers for early diagnosis of Mycobacterial infections are needed. Unlike previous studies, the current study focused on identifying biomarkers for JD from differentially expressed transcripts in whole blood of HD and LD MAP-inoculated calves at 6 and 9 months after inoculation with MAP.

In this study, key inflammation and immune-related genes (ALOX15, ALOX5AP, BNBD9-Like, GPR77, and S100A9) were downregulated 6 months after inoculation. ALOX15 belongs to lipoxygenases (LOXs) involved in metabolism and production of fatty acid hydroperoxidases [25]. ALOX5AP along with ALOX5 has a role in leukotriene biosynthesis, which is implicated in various inflammatory responses [26]. Enzyme 12/15-LOX or 15-LOX1 is activated by IL-4 in Th2 type response in monocytes and macrophages [27]; it has a role in resolution of inflammation [28,29] and synthesis of lipoxins, which also reduces inflammation by inhibiting chemotaxis, adhesion and superoxide generation [30]. Down-regulation of ALOX15 and ALOX5AP in the early stages of MAP infection correspond to the expected Th1 response-driven IFN-γ production that inhibits expression of LOXs genes. Proteins BNBD9-Like and GPR77 act as chemoattractants to immature dendritic cells [31,32], which function as professional antigen-presenting cells. S100A9 is a pro-inflammatory damage-associated molecular pattern (DAMPs) molecule secreted by phagocytes, granulocytes, monocytes and early differentiation stage macrophages. DAMPs are recognized by Toll-like receptors (TLRs), leading to their activation, which results in inflammation [33]. Down-regulation of these inflammatory and immune-related genes was consistent with a reduced capacity for an appropriate inflammatory response in MAP-infected animals.

Canonical pathway analysis revealed alterations in phosphoinositol biosynthesis and metabolism, and the JAK-STAT signalling pathway. Phosphatidylinositol signalling pathways are of interest in MAP infection, since these lipids act as chemoattractants and mediate immune cell migration [34]. Furthermore, *Mycobacterium* spp. is known to control host lipid metabolism to establish an infection [35]. Many genes involved in phosphatidylinositol signalling pathways were downregulated in this study. Conversely, the JAK-STAT pathway is the main signalling cascade involved in activation and regulation of host immune response following activation by cytokines and growth factors [36]. In this study, there were activation of STAT proteins and downregulation of other JAK-STAT association pathways such as Ras and MEK 1/2. Moreover, the negative regulator of the JAK-STAT pathway suppressors of cytokine signalling (SOCS) was upregulated and protein inhibitor of activated STATs (PIAS) was downregulated. The phosphoinositide 3-kinase (PI3K) pathway was also activated, leading to m-TOR mediated activation of STAT3. Therefore, we concluded that the overall JAK-STAT pathway was activated in MAP-inoculated calves 6 months after inoculation.

Lymphocyte and leukocyte migration were activated in the downstream pathway analysis. There was upregulation of chemokine (C-X-C motif) ligand 10 (CXCL10), SH2 domain containing 1A (SH2D1A), TXK tyrosine kinase (TXK); they enable lymphocyte activation and migration [37-39]. The latter was consistent with the expected movement of leukocyte and MAP antigen-activated lymphocytes to the site of inflammation or infection.

At 6 months after inoculation, key immune functions such as macrophage response and phagocytosis migration of granulocytes and phagocytosis by other antigen presenting cells were downregulated. This was in agreement with MAP-induced inhibition of macrophage maturation described in other studies [14,40]. Necrosis and apoptosis of cells were concurrently inhibited, as reported at 3 months after inoculation [12] and also in other studies [41-43] as a virulence mechanism of MAP to subvert immune detection and immune escape.

Interestingly, genes that would facilitate failure of growth were downregulated, as reported 3 month after MAP inoculation [12]. This suggests a response counteracting malabsorption, due to early host immune response and inflammation which manifested as histological and gross pathological lesions in MAP-infected tissues [13], as a homeostatic mechanism to support growth of the animal.

CD40L was activated based on upstream analysis done using differentially expressed transcripts (6 months after inoculation) between HD, LD and control. CD40L is expressed on CD4^+^ T cells; it interacts with CD40 on the B cells, and activates them to produce a B cell response, immunoglobulin class switch and activation of macrophages to produce IFN-γ [44]. It is noteworthy that 3 of the 5 HD animals in this study had seroconverted 6 months after inoculation. Based on upstream analysis, TGF-beta1 and miRNAs like let-7a-5p and miR-16-5p were suggested to be inhibited at 6 months after inoculation. TGF-beta1 is an anti-inflammatory, pro-fibrotic and macrophage deactivating cytokine, implicated in the pathogenesis of many intracellular pathogens [45,46]. *Mycobacterium avium* complex (MAC) pathogens induce many cytokines, especially TGF-beta1 [47,48]. TGF-beta1 has an important role in granulomatous infection; its levels are related to intracellular replication of MAC [49]. Future studies analysing TGF-beta1 in subclinical and clinical animals might help to validate it as biomarker for MAP diagnosis and to understand its role in immunopathogenesis.

Micro RNAs regulate expression of many genes by post-translation modification of mRNAs and are associated with cancer and infectious disease [50-52]. In Crohn’s disease (CD) patients, several miRNAs (e.g.miR-16 and miR-23b) were associated with active and chronically active CD patients [53,54]. In the current study, miR-2277-3p was activated, whereas several other miRNAs were inhibited. The exact role of these miRNAs is not known, but they have potential as markers for JD.

At 9 months after inoculation, gene expression of genes was generally repressed; based on ANOVA, there were only 80 transcripts differentially expressed among HD, LD and control calves (*p <* 0.05). Similarly, in previous translational studies [55,56] on gene expression in macrophages and PBMC’s infected with MAP, the number of differentially expressed genes decreased with time after inoculation.

BOLA/MHC-1 was downregulated 9 months after inoculation, in agreement with a previous study [57]. BOLA is a key antigen-presenting protein that carries intracellular proteins to the cell membrane and presents it to cytotoxic T cells for detection and eventual control or killing of intracellular pathogens. In addition, IL4R and CCR7 were also downregulated. IL4R and CCR7 belong to G-protein coupled receptors present on B and T lymphocytes and leukocytes, enabling these cells to move to sites of inflammation and secondary lymphoid organs [58,59].

Autophagy, antigen presenting cells, cytotoxicity of T-lymphocytes and organisation of the cytoplasm for cell replication were all activated 9 months after inoculation. The role of autophagy in *Mycobacterial* infections has been reported [55,60]; furthermore, we reported that autophagy was already activated 3 months after inoculation [12]. Perhaps autophagy acts as a compensatory mechanism to present intracellular MAP antigens in the context of reduced antigen presentation by MHC-1/BOLA.

Canonical pathway analysis revealed differentially expressed genes in MAP-infected calves to be involved in IL-4 signalling pathway. IL-4 is a multifunctional cytokine produced by CD4^+^ Th2 cells, basophils and mast cells. In this study, there was upregulation of son of sevenless (SOS), 1-phosphotidylionositol 3-phosphate, P70 S6 kinase (P70S6K) and nuclear factor of activated T cells (NFAT) belonging to alternate IL-4 signalling pathway. However, IL-4R in the classical IL-4 signalling pathway was downregulated. IL-4 is important for granuloma formation in Mycobacterial infection and defense against Mycobacterial infection [61]. At 9 months after inoculation, not many genes were differentially expressed; consequently, upstream analysis did not lead to many predictions. Activation of miR-22-3p, miR-4283, miR-320b, miR-3175 and few other miRNAs are listed in Additional file 6, according to upstream analysis at 9 months after inoculation.

BOLA and BNBD9-Like genes were downregulated and CD46 gene was upregulated in both LD and HD calves for the duration of the trial (3 to 15 months after inoculation). Identification of genes that are consistently differentially expressed genes warrants a larger cohort study to validate these genes as potential biomarkers for MAP infection. In this study, BNBD9-LIKE and BOLA genes were differentially expressed 6 and 9 months after inoculation. The ubiquitously expressed transmembrane glycoprotein CD46 was upregulated 3 months after inoculation [12]. It prevents unwanted complement-mediated killing of cells by acting as a cofactor in factor-I mediated degradation of C3b and C4b opsonins [62]. Although CD46 has a regulatory role in Th1 responses [63,64], it also interacts with STE20/SPS1-related proline/alanine-rich kinase (SPAK) kinase and E-cadherin, which have important role sin maintaining epithelial barrier function in intestinal epithelial cells [65].

A large proportion of pathways, including immune and inflammation pathways, were consistently affected at 3, 6 and 9 months after inoculation, with relatively few pathways being affected at only a single time point. However, no obvious evolution was noticed in the affected pathways over the course of this follow-up.

In conclusion, many genes were differentially expressed between MAP-infected animals and control animals at 6 and 9 months after inoculation. BOLA, BNBD9-Like and CD46 were differentially expressed in both LD and HD calves as early as 3 months after inoculation until at least 15 months after inoculation. Dose of infection had an influence on the fold change of differentially expressed genes. Migration and trafficking of leukocytes and lymphocytes were activated, but there was more downregulation of immune response by inhibition of phagocytosis, downregulation of antigen presentation, macrophage phagocytosis and response. Furthermore, MHC-1/BOLA was downregulated and autophagy was activated, potentially compensating for any loss in antigen presentation by PRRs on the account of downregulation of MHC-1/BOLA. Finally, we propose that CD46, BNBD9-Like and BOLA are further considered as potential biomarkers for diagnosis of JD.

## Additional files

Additional file 1
**Differentially expressed genes at 6 months after infection.** Affymetrix probe ID, Symbol, Gene description. Additional file 2
**List of canonical pathways associated with differentially expressed genes at 6 months after infection.** Ingenuity Canonical Pathway, −log(*p*-value), Ratio, Molecules. Additional file 3
**List of upstream regulators and their targets in the dataset obtatined at 6 months after infection.** Upstream Regulator, Molecule Type, Predicted Activation State, Activation z-score, Target molecules in dataset. Additional file 4
**Differentially expressed genes at 9 months after infection.** Affymetrix probe ID, Symbol, Gene description. Additional file 5
**List of canonical pathways associated with differentially expressed genes at 9.** Ingenuity Canonical Pathway, −log(*p*-value), Ratio, Molecules. Additional file 6
**List of upstream regulators and their targets in differentially expressed genes at 9 months after infection.** Upstream Regulator, Molecule Type, Predicted Activation State, Activation z-score, Target molecules in dataset.
